# *In Vitro* Antilisterial Properties of Crude Methanol Extracts of *Garcinia kola* (Heckel) Seeds

**DOI:** 10.1100/2012/694828

**Published:** 2012-08-13

**Authors:** Dambudzo Penduka, Anthony I. Okoh

**Affiliations:** Applied and Environmental Microbiology Research Group (AEMREG), Department of Biochemistry and Microbiology, University of Fort Hare, Alice Private Bag X1314, Alice 5700, South Africa

## Abstract

Crude methanol extracts of *Garcinia kola* (Heckel) seeds were screened for their antilisterial activities against 42 *Listeria* bacteria isolated from wastewater effluents. The extract had activity against 45% of the test bacteria and achieved minimum inhibitory concentrations (MICs) ranging between 0.157 and 0.625 mg/mL. The rate of kill of the extract was determined against four representative *Listeria* species in the study, and the results showed that the highest percentage of bacteria cells were killed after the maximum exposure time of 2 h at the highest concentration of 4 × MIC value, with the maximum number of bacteria cells killed being for *L. ivanovii* (LEL 30) 100%, *L. monocytogenes* (LAL 8) 94.686%, *L. ivanovii* (LEL 18) 60.330%, and *L. grayi* (LAL 15) 56.071% We therefore conclude that the nature of inhibition of the crude methanol extracts of *Garcinia kola* seeds can be either bactericidal or bacteriostatic depending on the target *Listeria* species and can also differ among same species as evidenced by *L. ivanovii* strains LEL 30 and LEL 18.

## 1. Introduction

Medicinal plant remedies are an integral part of the history and culture of people in developing countries where they are widely used to cover basic health care needs and their use is also becoming part of the integrative healthcare system of developed nations as complementary and alternative medicines [1]. Plant remedies are favoured as a cheaper and readily available alternative form of treatment and Borris [2] estimated that between 250000 and 500000 plant species exist on earth thereby representing a biologically and chemically diverse resource.

Plants also are a source of many new useful phytochemicals of great diversity, which have inhibitory effects on different types of microorganisms *in vitro* [1]. Examples of these phytochemicals include terpenoids, essential oils, alkaloids, lectins, polypeptides, polyacetylenes, and phenolics. Phenolics can be subdivided into phenolic acids, flavonoids, quinones, tannins, coumarins, and simple phenols [3]. These antimicrobial compounds from plants also have potential to inhibit bacteria through different mechanisms other than those presently used by antibiotics and this may have clinical value in treatment of resistant microbial strains [4].

In this connection *Listeria* species is one such bacteria with increasing reports of its resistance to conventional antibiotics [5–8]. *Listeria* species are Gram-positive bacteria that are widespread in nature and they have been recovered from raw vegetables, raw milk, fish, poultry, and meats, such that infection most likely begins following ingestion of the organism in contaminated food, and clinical manifestations of the invasive listeriosis are usually severe and may include abortion, sepsis, and meningoencephalitis [9].

*Listeria* crosses the mucosal barrier of the intestine and, once in the bloodstream, may disseminate hematogenously to any site although the liver is thought to be the first target organ, where active multiplication occurs until cell-mediated immune response gains control of the infection [9, 10]. In healthy individuals the continual exposure to listerial antigens may result in maintenance of antilisteria memory T cells; however in immune-compromised individuals this exposure may result in prolonged bacteremia and progress to overt listeriosis such that approximately 70% of nonperinatal *Listerial* infections occur in individuals with malignancies, AIDS, organ transplants, or in those receiving corticosteroid therapy [9]. The case-fatality rates of *Listeria* infection vary from country to country, but invariably the highest mortality is among newborns (25%–50%) due to infection acquired from their mothers, whilst mortality among those over 60 years of age is also high ranging between 10%–20% [11].

Given the high mortality rates of *Listeria* infection against a background of antibiotic resistant strains it becomes imperative to explore for alternative forms of treatment, and having acknowledged the medical importance of plant remedies and their potential in curbing antibiotic resistance this study therefore focuses on the antilisterial activities of the crude methanol extract of *Garcinia kola* seeds. *Garcinia kola* is a traditional medicinal plant which has been used since time immemorial for its medicinal purposes mainly in its indigenous origins of central and West Africa [12]. Almost every part of the plant has been found to be of medical importance; the nut is used for nervous alertness, induction of insomnia and also as a masticatory; the root of the plant is used as bitter chew sticks; the stem bark is used as a purgative; the latex is externally applied to fresh wounds to prevent sepsis, thereby assisting in wound healing [13]. Various studies have focused on the antimicrobial and therapeutic potentials of *Garcinia kola* plant [14–18], but despite all that, there is still paucity of information on the antilisterial activities of the crude methanol extract of the seeds.

## 2. Materials and Methods

### 2.1. Plant Material

The ground seed powder of *Garcinia kola* was obtained from the plant material collection of the Applied and Environmental Microbiology Research Group (AEMREG) laboratory, University of Fort Hare Alice. South Africa.

### 2.2. Preparation of Extracts

The extracts were prepared following the descriptions of Basri and Fan [19]. A 100-gram measurement of the seed powder was steeped in 500 mL of methanol solvent for a 48 h period with shaking in an orbital shaker (Stuart Scientific Orbital Shaker, UK). The resultant extract was centrifuged at 3000 rpm for 5 min at 4°C (Beckman Model TJ-6RS Centrifuge, Great Britain), the supernatant was then filtered through Whatman No.1 filter paper, while the residue was then used in the second extraction process involving 300 mL of the solvent. The combined extracts were concentrated using a rotary evaporator at 65°C (Steroglass S.R.L, Italy), after which they were dried to a constant weight under a stream of air in a fume cupboard at room temperature. Dimethyl sulphoxide (DMSO) at a concentration equal to 5% of the total volume which was made up with sterile distilled water was used to aid the reconstitution of the dried extract when making test concentrations.

### 2.3. Test *Listeria *Strains

The 42 test *Listeria* isolates used in this study were obtained from the culture collection of the Applied and Environmental Microbiology Research Group (AEMREG) laboratory at the University of Fort Hare, Alice, South Africa. The bacteria were previously isolated from wastewater effluents in the Eastern Cape Province of South Africa and belonged to three species groups which are *L. ivanovii*, *L. grayi*, and* L. monocytogenes* [20].

### 2.4. Preparation of the Inoculum 

The EUCAST [21] colony suspension method was used to prepare the inoculums of the test organisms. In brief, colonies picked from 24 h old cultures were suspended in saline solution (0.85% NaCl) to give an optical density of approximately 0.1 at 600 nm after which the suspension was then diluted a hundredfold before use.

### 2.5. Antibacterial Susceptibility Test

The agar well diffusion method according to Irobi et al. [22] with modifications was used to determine the sensitivity of the test *Listeria* to the extract. The prepared bacterial suspension (100 *μ*L) was inoculated into sterile molten Mueller-Hinton agar medium at 50°C in a MacCarthney bottle, mixed gently, and then poured into a sterile petri dish and allowed to solidify. A sterile 6 mm diameter cork borer was used to bore wells into the agar medium after which the wells were filled up with approximately 100 *μ*L of 10 mg/mL extract solution. The plates were then allowed to stand on the laboratory bench for 1 h to allow proper diffusion of the extract into the medium before incubation at 37°C for 24 h, and thereafter the zones of inhibition were measured. Ciprofloxacin (2 *μ*g/mL) was used as a positive control, and sterile distilled water was used as the negative control whilst 5% DMSO was also tested to determine its effect on each organism.

### 2.6. Determination of the Minimum Inhibitory Concentration (MIC) and Minimum Bactericidal Concentration (MBC)

The MICs of the extract against the susceptible *Listeria* isolates were determined using the broth microdilution assay method of EUCAST [21] and carried out in sterile disposable flat-bottomed 96-well microtiter plates. Twofold serial dilutions using sterile distilled water were carried out from 10 mg/mL of the stock plant extract to make 9 test concentrations ranging from 0.039 to 10 mg/mL. Double strength Mueller-Hinton broth (100 *μ*L) was introduced into all the 96 wells and 50 *μ*L of the varying test concentrations of the extracts were added in decreasing order along with 50 *μ*L of the test organism suspension. Column 1 was used as the sterility wells containing 100 *μ*L of sterile distilled water in addition to the 100 *μ*L of Mueller-Hinton broth, column 2 was used as the positive control wells containing 100 *μ*L of the broth, 50 *μ*L of ciprofloxacin, and 50 *μ*L of the test organism, column 3 was used as the negative control wells containing 100 *μ*L of the broth, 50 *μ*L sterile distilled water, and 50 *μ*L of the test organism whilst columns 4 to 12 were used as test wells containing 100 *μ*L of the broth, 50 *μ*L of the test extract concentration, and 50 *μ*L of the test organism. The plates were then incubated at 37°C for 18–24 h. Results were read visually by adding 40 *μ*L of 0.2 mg/mL of *ρ*-iodonitrotetrazolium violet (INT) dissolved in sterile distilled water into each well [4]. A pinkish coloration is indicative of microbial growth because of their ability to convert INT to red formazan [23]. The MIC was recorded as the lowest concentration of the extract that prevented the appearance of visible growth of the organism after 24 h of incubation [21].

The method of Sudjana et al. [24] was used to determine the MBC from the MIC broth microdilution assays through subculturing 10 *μ*L volumes from each well that did not exhibit growth after 24 h of incubation and spot inoculating it onto fresh Mueller-Hinton agar plates. The plates were incubated for 48 h after which the numbers of viable colonies were counted. The MBC was defined as the lowest concentration killing more than or equal to 99.9% of the inoculum compared with initial viable counts [24].

### 2.7. Rate of Kill Assay

The time-kill assay was done according to the method of Odenholt et al. [25] following the descriptions of Akinpelu et al. [26]. The selected test *Listeria* isolates, namely, *L. ivanovii *(LEL 18)*, L. grayi *(LAL 15), *L. monocytogenes *(LAL 8), and *L. ivanovii* (LEL 30), were used for the rate of kill studies as representatives of the *Listeria* species used in the study. The turbidity of the 18 h old test *Listeria* was first standardized to 10^8^ cfu/mL. Four different concentrations of the plant extract were made starting from the MIC to 4 × MIC value obtained against each test organism. A 0.5 mL volume of the organism suspension was added to 4.5 mL of the different extract's concentrations, held at room temperature and the rate of kill determined over a period of 2 h. Exactly 0.5 mL volume of each suspension was withdrawn at 15-minute intervals and transferred to 4.5 mL of nutrient broth recovery medium containing 3% “Tween 80” to neutralize the effects of the antimicrobial compound carryovers on the test organisms [26]. The suspension was then serially diluted and 0.5 mL was plated out for viable counts using the pour plate method. The plates were thereafter incubated at 37°C for 48 h. The control plates contained the test organism without the plant extracts. The emergent colonies were counted and compared with the counts of the culture control. 

### 2.8. Statistical Analysis

The SPSS 19.0 version for windows program was used to determine the means and standard deviations of the zones of inhibitions, with the one-way analysis of variance (ANOVA) of the same program being used to determine the means and standard deviations of the rate of kill results.

## 3. Results

### 3.1. Antibacterial Susceptibility Tests

Table 1 shows the zones of inhibitions observed against the susceptible *Listeria* isolates. The methanol extract was active against 19 of the 42 test *Listeria* used in the study giving a percentage activity of 45%. The highest zone of inhibition was against *L. ivanovii* (LDB 7) with a zone of inhibition of 19 mm, the lowest zone was 10 mm observed against 7 isolates, namely, *L. ivanovii* (LDB 11), *L. ivanovii* (LEL 9),* L. ivanovii* (LAL 9), *L. grayi* (LAL 12), *L. grayi* (LAL 15), *L. ivanovii* (LDB 3), and *L. ivanovii* (LAL 11). The positive control ciprofloxacin was active against all the 42 isolates whilst the negative control (sterile distilled water) and 5% DMSO were both not active against any of the isolates.

### 3.2. Minimum Inhibitory Concentration and Minimum Bactericidal Concentration

The results of the MICs and MBCs of the extract against the susceptible *Listeria* isolates are shown in Table 2. The MICs ranged from 0.157–0.625 mg/mL, with MIC values of 0.157 mg/mL and 0.625 mg/mL being recorded against 5 isolates each whilst the MIC value of 0.313 mg/mL was observed against 9 isolates only. The MBCs ranged between 5 and 10 mg/mL with the extract's lowest MBC value of 5 mg/mL being recorded against 3 isolates, namely, *L. ivanovii *(LEL 30), *L. ivanovii *(LDB 12), and *L. ivanovii* (LDB 10), whilst an MBC value of 10 mg/mL was observed against the rest of the isolates. The overall mean MIC and MBC values of the extract were 0.354 mg/mL and 9.211 mg/mL, respectively, against the 19 test strains.

### 3.3. Rate of Kill Assay

**Figure 1:**
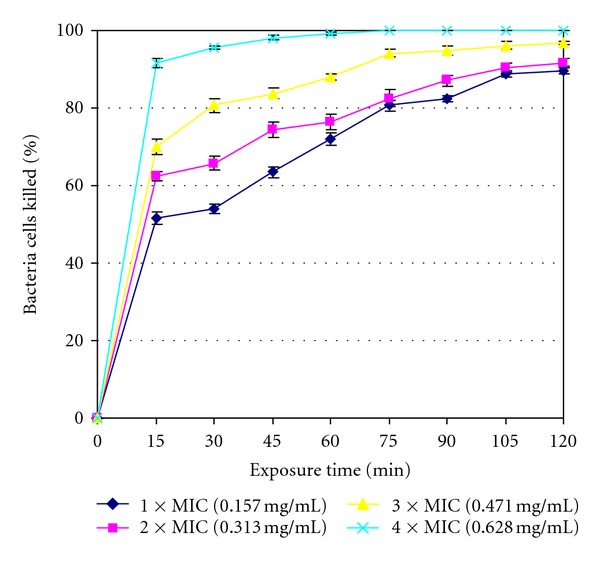
Rate of kill profile for *L. ivanovii* (LEL 30) by crude methanol extracts of *Garcinia kola* seeds.

**Figure 2:**
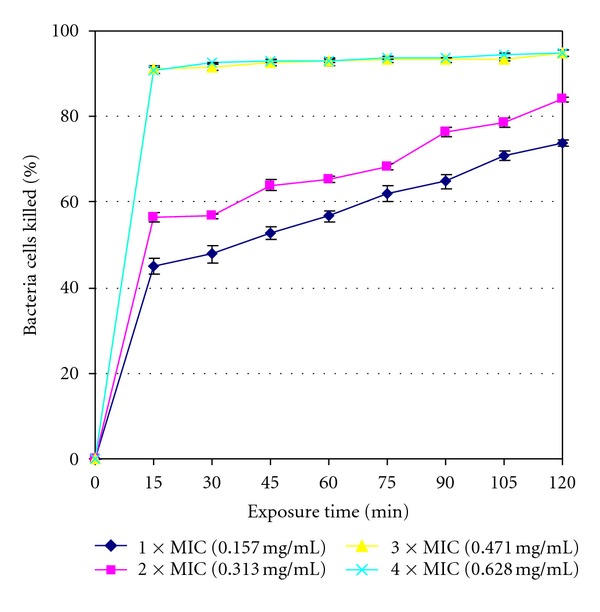
Rate of kill profile for *L. monocytogenes* (LAL 8) by crude methanol extracts of *Garcinia kola *seeds.

**Figure 3:**
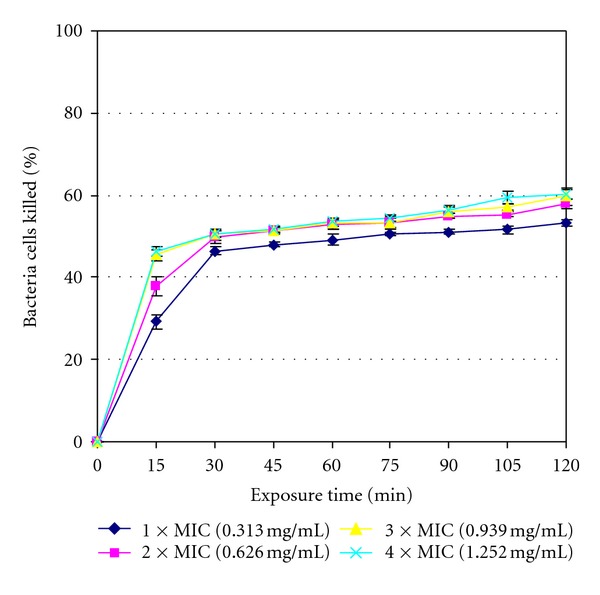
Rate of kill profile for *L. ivanovii* (LEL 18) by crude methanol extracts of *Garcinia kola* seeds.

**Figure 4:**
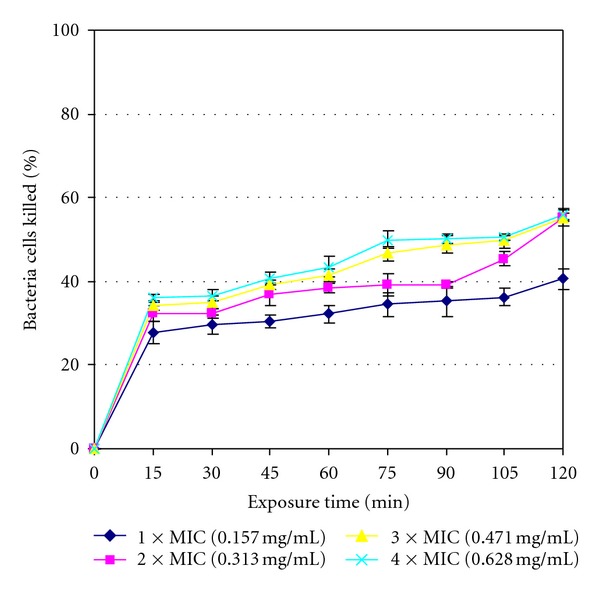
Rate of kill profile for *L. grayi* (LAL 15) by crude methanol extracts of *Garcinia kola* seeds.

The rates of kill results are shown in Figure 1 to Figure 4 for the four isolates tested with the standard deviations also being included in the curves. The extract was bactericidal against *L. ivanovii* (LEL 30) as it killed all (100%) of the initial bacterial population at 75 min exposure time at 4 × MIC value as shown in Figure 1. The extract was however bacteriostatic after the maximum exposure time of 2 h at the highest concentration of 4 × MIC value against the other three isolates managing to kill 94.686% bacteria cells against *L. monocytogenes* (LAL 8) (Figure 2), 60.330% bacteria cells against *L. ivanovii* (LEL 18) (Figure 3), and 56.071% bacteria cells against *L. grayi* (LAL 15) (Figure 4).

## 4. Discussion

This study revealed the antilisterial activities of the methanol extract of *Garcinia kola* seeds. The extract was active against each *Listeria* species used in the study and had a 45% activity. Similarly the methanol extract of *Garcinia kola* seeds in other studies has been found to be also active against other Gram-positive bacteria such as *Staphylococcus aureus*, *Streptococcus pneumonia* [14], and *Staphylococcus sciuri *[27].

The extract's MIC values against the test *Listeria* bacteria ranged from 0.157–0.625 mg/mL. A separate study involving the methanol extract of *Garcinia kola* seeds against Gram-positive bacteria also showed MIC values within the same range as those observed in this study; the findings of Sibanda et al. [27] showed that the extract had an MIC value of 0.312 mg/mL against all four *Staphylococcus* strains tested. However findings involving Gram-negative bacteria revealed higher MIC ranges, Penduka et al.'s [28] study involving *Vibrio* isolates revealed higher MIC ranges with values ranging from 0.313 to 2.5 mg/mL whilst that by Nwaokorie et al. [29] involving *Fusobacterium nucleatum *species showed MIC values ranging from 1.25 to 12.50 mg/mL, and the higher MIC values against Gram-negative bacteria could be due to the presence of the outer membrane present in Gram-negative bacteria which acts as a barrier against antibiotics that work inside the cell, a factor attributing to antibiotic resistance. 

The highest number of bacteria cells killed was achieved at the highest concentration of 4 × MIC and at the maximum exposure time of 2 h for all the four organisms. The extract was bactericidal against *L. ivanovii *(LEL 30) only but was however bacteriostatic against the other three *Listeria* isolates since by definition a more than or equal to 99.9% killing rate is characteristic of a cidal agent whilst a lower killing rate is characteristic of a bacteriostatic agent [30]. The results show that the methanol extract can be either bactericidal or bacteriostatic depending on the *Listeria* isolate tested and the rate of kill can vary even within same species isolates as evidenced by *L. ivanovii* isolates LEL 30 and LEL 18 in this study. 

*Garcinia kola* seeds methanol extract's rate of kill studies by Penduka et al. [28] involving *Vibrio* isolates similarly showed the highest number of bacteria cells killed being achieved at the highest concentration of 4 × MIC after maximum exposure time of 2 h; however only a bacteriostatic nature of inhibition was noted in the study, whilst in a study by Nwaokorie et al. [29] involving *Fusobacterium nucleatum* clinical isolates and a biofilm produced by the association of *Aggregatibacter actinomycetemcomitans*, *Porphyromonas gingivalis, Prevotella intermedia*, and *Fusobacterium nucleatum* isolates at 100 mg/mL concentration, showed a bactericidal activity by killing the entire bacterial population after 1 h exposure time. A point to note however in the study by Nwaokorie et al. [29] is the concentration of the extract used in the study which was 4 × MIC value against the biofilm and 8 × MIC or 80 × MIC value against the *Fusobacterium nucleatum* clinical isolates and this achieved a bactericidal effect; this can therefore mean that an increase in the concentration of the seed's methanol extract to MIC levels above the 4 × MIC value can result in bactericidal activity of the extract at minimum exposure time. 

The methanol solvent is known to extract a wide range of phytochemicals such as anthocyanins, terpenoids, saponins, tannins, xanthoxylines, totarol, quassinoids, lactones, flavones, phenones, and polyphenols [3]. Some of these phytochemicals such as flavonoids, tannins, cardiac glycosides, saponins, steroids, and reducing sugars have also been found to be present in *Garcinia kola* seeds [31]. Flavones are a class of flavonoids of which flavonoids are well known for possessing a wide range of therapeutic properties such as antioxidant, antipyretic (fever-reducing), analgesic, and spasm inhibiting properties [32], in addition to possessing antibacterial, antiviral, antiallergic, and anti-inflammatory activities [33]. Saponins have been reported to have antifungal properties [34] and tannins are known to posses antiviral, antibacterial, and antitumor activities [35], such that either of these phytochemicals could have been responsible for the observed antibacterial activities of the extract in this study. 

The results found in this study are therefore good preliminary findings that are good foundations for the further isolation and characterisation of the antilisterial compounds in *Garcinia kola* seeds as the purified active compound could be more potent in comparison to the crude extract and these are subjects of ongoing studies in our research group.

## Figures and Tables

**Table 1:** Zones of inhibition including the standard deviations of ciprofloxacin and the crude methanol extract of *Garcinia kola* seeds against *Listeria *isolates.

Test organisms	Zones of inhibition (mm)		Test organisms	Zones of inhibition (mm)	
	Meth	Cipro		Meth	Cipro
*L. grayi* (LAL 13)	0	20 ± 3.055	*L. ivanovii* (LEL 18)	12 ± 0.577	20 ± 3.215
*L. ivanovii* (LEL 17)	11 ± 1.155	19 ± 1.528	*L. ivanovii* (LEL 29)	0	8 ± 0.577
*L. ivanovii *(LEL 30)	15 ± 0.577	30 ± 0.577	*L. ivanovii* (LEL 15)	0	13 ± 2.082
*L. ivanovii* (LDB 11)	10 ± 1.155	20 ± 1	*L. ivanovii* (LDB 9)	13 ± 1.155	25 ± 2.082
*L. ivanovii* (LEL9)	10 ± 2.517	16 ± 2.082	*L. ivanovii* (LDB 10)	15 ± 1.155	25 ± 0.577
*L. ivanovii* (LEL 1)	17 ± 0.577	17 ± 0.577	*L. ivanovii* (LEL 2)	0	28 ± 1.528
*L. ivanovii* (LEL 5)	0	11 ± 0.577	*L. ivanovii* (LEL 6)	0	11 ± 1.732
*L. ivanovii* (LEL 3)	0	35 ± 3.055	*L. ivanovii* (LEL 4)	0	14 ± 1
*L. ivanovii* (LEL 19)	0	25 ± 4.041	*L. ivanovii* (LEL 10)	0	20 ± 2.082
*L. ivanovii* (LAL 9)	10 ± 0.577	25 ± 1.732	*L. ivanovii* (LAL 11)	10 ± 1.732	17 ± 2.646
*L. grayi* (LAL 12)	10 ± 1.528	17 ± 1.155	*L. ivanovii* (LAL 10)	11 ± 2.082	15 ± 2.082
*L. grayi* (LAL 15)	10 ± 2	18 ± 2.082	*L. ivanovii* (LAL 14)	0	30 ± 2.517
*L. ivanovii* (LDB 1)	0	15 ± 2.082	*L. ivanovii* (LDB 2)	0	14 ± 0
*L. ivanovii* (LAL 6)	0	19 ± 1.155	*L. ivanovii* (LAL 5)	0	20 ± 1.528
*L. ivanovii* (LAL 7)	0	20 ± 1.528	*L. monocytogenes* (LAL 8)	18 ± 1.528	12 ± 1
*L. ivanovii* (LDB 7)	19 ± 0.577	27 ± 0.577	*L. ivanovii* (LDB 12)	14 ± 1.155	25 ± 1.528
*L. ivanovii* (LDB 3)	10 ± 0.577	15 ± 1	*L. ivanovii* (LDB 8)	0	20 ± 1.732
*L. ivanovii* (LEL 7)	0	9 ± 1	*L. ivanovii* (LEL 8)	0	30 ± 1.528
*L. ivanovii* (LEL 14)	0	35 ± 2	*L. ivanovii* (LEL 16)	11 ± 0.577	15 ± 1.528
*L. grayi* (LAL 3)	0	13 ± 3.055	*L. ivanovii* (LAL 4)	0	20 ± 2
*L. ivanovii* (LAL 2)	11 ± 1.155	16 ± 1	*L. ivanovii* (LAL 1)	0	20 ± 2

Note. *Meth* denotes methanol extract; *Cipro* denotes ciprofloxacin; number ± number denotes zone of inhibition in mm ± standard deviation in mm whereby each observation is a product of 3 replicate experiments; mm denotes millimeters.

**Table 2:** Minimum inhibitory concentrations (MICs) and minimum bactericidal concentrations (MBCs) of crude methanol extracts of *Garcinia kola* (Heckel) seeds against susceptible *Listeria* isolates.

Organism	Methanol Extracts	
	MIC (mg/mL)	MBC (mg/mL)
*L. ivanovii* (LEL 9)	0.313	10
*L. ivanovii* (LEL 18)	0.313	10
*L. ivanovii* (LAL 10)	0.625	10
*L. ivanovii* (LEL 30)	0.625	5
*L. ivanovii* (LEL 16)	0.313	10
*L. monocytogenes* (LAL 8)	0.157	10
*L. ivanovii* (LDB 12)	0.625	5
*L. ivanovii* (LDB 10)	0.313	5
*L. ivanovii* (LEL 1)	0.625	10
*L. ivanovii* (LAL 11)	0.157	10
*L. ivanovii* (LDB 3)	0.313	10
*L. grayi* (LAL 15)	0.157	10
*L. grayi* (LAL 12)	0.313	10
*L. ivanovii* (LDB 11)	0.313	10
*L. ivanovii* (LAL 2)	0.157	10
*L. ivanovii* (LEL 17)	0.313	10
*L. ivanovii* (LDB 7)	0.313	10
*L. ivanovii* (LDB 9)	0.625	10
*L. ivanovii* (LAL 9)	0.157	10

Note. mg/mL denotes milligrams per milliliter.

## References

[1] Arif T, Bhosale JD, Kumar N (2009). Natural products-antifungal agents derived from plants. *Journal of Asian Natural Products Research*.

[2] Borris RP (1996). Natural products research: perspectives from a major pharmaceutical company. *Journal of Ethnopharmacology*.

[3] Cowan MM (1999). Plant products as antimicrobial agents. *Clinical Microbiology Reviews*.

[4] Eloff JN (1998). A sensitive and quick microplate method to determine the minimal inhibitory concentration of plant extracts for bacteria. *Planta Medica*.

[5] Abuin CMF, Fernandez EJQ, Sampayo CF, Otero JLR, Rodriguez LD, Saez AC (1994). Susceptibilities of *Listeria* species isolated from food to nine antimicrobial agents. *Antimicrobial Agents and Chemotherapy*.

[6] Arslan S, Özdemir F (2008). Prevalence and antimicrobial resistance of *Listeria spp.* in homemade white cheese. *Food Control*.

[7] Chen BY, Pyla R, Kim TJ, Silva JL, Jung YS (2010). Antibiotic resistance in *Listeria* species isolated from catfish fillets and processing environment. *Letters in Applied Microbiology*.

[8] Nwachukwu NC, Orji FA, Iheukwumere I, Ekeleme UG (2010). Antibiotic resistant environmental isolates of *Listeria monocytogenes* from anthropogenic lakes in Lokpa-Ukwu, Abia State of Nigeria. *Australian Journal of Basic and Applied Sciences*.

[9] Snapir YM, Vaisbein E, Nassar F (2006). Low virulence but potentially fatal outcome-*Listeria ivanovii*. *European Journal of Internal Medicine*.

[10] Vázquez-Boland JA, Kuhn M, Berche P (2001). *Listeria* pathogenesis and molecular virulence determinants. *Clinical Microbiology Reviews*.

[11] Bortolussi R (2008). Listeriosis: a primer. *CMAJ*.

[12] Iwu MM (1993). *Handbook of African Medicinal Plants*.

[13] Uko OJ, Usman A, Ataja AM (2001). Some biological activities of *Garcinia kola* in growing rats. *Veterinarski Arhiv*.

[14] Adeleke OE, Ojo OP, Idowu PA (2006). Antibacterial activity of methanolic extract of *Garcinia kola* (Heckel) seeds and standard antibiotics. *African Journal of Clinical and Experimental Microbiology*.

[15] Tebekeme O, Prosper AE (2007). *Garcinia kola* extract reduced cisplatin-induced kidney dysfunction in rats. *African Journal of Biochemistry Research*.

[16] Ogbulie JN, Ogueke CC, Nwanebu FC (2007). Antibacterial properties of *Uvaria chamae, Congronema latifolium, Garcinia kola, Vemonia amygdalina*, and *Aframomium melegueta*. *African Journal of Biotechnology*.

[17] Adegbehingbe OO, Adesanya SA, Idowu TO, Okimi OC, Oyelami OA, Iwalewa EO (2008). Clinical effects of *Garcinia kola* in knee osteoarthritis. *Journal of Orthopaedic Surgery and Research*.

[18] Penduka D, Okoh AI (2011). In-Vitro antagonistic characteristics of crude aqueous and methanolic extracts of *Garcinia kola* (Heckel) seeds against some *Vibrio* bacteria. *Journal of Medicinal Plants Research*.

[19] Basri DF, Fan SH (2005). The potential of aqueous and acetone extracts of galls of *Quercus infectoria* as antibacterial agents. *Indian Journal of Pharmacology*.

[20] Odjadjare EEO, Obi LC, Okoh AI (2010). Municipal wastewater effluents as a source of *Listerial* pathogens in the aquatic milieu of the eastern cape province of South Africa: a concern of public health importance. *International Journal of Environmental Research and Public Health*.

[21] European committee for Antimicrobial Susceptibility Testing (2003). Determination of minimum lnhibitory Concentration (MICs) of antimicrobial agents by broth dilution. *Clinical Microbiology and Infection*.

[22] Irobi ON, Moo-Young M, Anderson WA (1996). Antimicrobial activity of Annatto (*Bixa orellana*) extract. *International Journal of Pharmacognosy*.

[23] Iwalewa EO, Suleiman MM, Mdee LK, Eloff JN (2009). Antifungal and antibacterial activities of different extracts of *Harungana madagascariensis* stem bark. *Pharmaceutical Biology*.

[24] Sudjana AN, D’Orazio C, Ryan V (2009). Antimicrobial activity of commercial *Olea europaea* (olive) leaf extract. *International Journal of Antimicrobial Agents*.

[25] Odenholt I, Löwdin E, Cars O (2001). Pharmacodynamics of telithromycin in vitro against respiratory tract pathogens. *Antimicrobial Agents and Chemotherapy*.

[26] Akinpelu DA, Adegboye MF, Adeloye OA, Okoh AI (2008). Biocidal activity of partially purified fractions from methanolic extract of *Garcinia kola* (Heckel) seeds on bacterial isolates. *Biological Research*.

[27] Sibanda T, Olaniran AO, Okoh AI (2010). In vitro antibacterial activities of crude extracts of *Garcinia kola* seeds against wound sepsis associated *Staphylococcus* strains. *Journal of Medicinal Plant Research*.

[28] Penduka D, Okoh OO, Okoh AI (2011). In-vitro antagonistic characteristics of crude aqueous and methanolic extracts of *Garcinia kola* (Heckel) seeds against some *Vibrio* bacteria. *Molecules*.

[29] Nwaokorie F, Coker A, Ogunsola F (2010). Antimicrobial activities of *Garcinia kola* on oral *Fusobacterium nucleatum* and biofilm. *African Journal of Microbiology Research*.

[30] Pankey GA, Sabath LD (2004). Clinical relevance of bacteriostatic versus bactericidal mechanisms of action in the treatment of gram-positive bacterial infections. *Clinical Infectious Diseases*.

[31] Adegboye MF, Akinpelu DA, Okoh AI (2008). The bioactive and phytochemical properties of *Garcinia kola* (Heckel) seed extract on some pathogens. *African Journal of Biotechnology*.

[32] Krishnaiah D, Devi T, Bono A, Sarbatly R (2009). Studies on phytochemical constituents of six Malaysian medicinal plants. *Journal of Medicinal Plant Research*.

[33] Cook NC, Samman S (1996). Flavonoids-Chemistry, metabolism, cardioprotective effects, and dietary sources. *Journal of Nutritional Biochemistry*.

[34] Sodipo OA, Awanji MA, Kolawole FB, Oduntuga AA (1991). Saponin is the active fungal principle in *Garcinia kola*, Heckle seed. *Bioscience Research Communications*.

[35] Kunle OF, Egharevba HO (2009). Preliminary studies on *Vernonia ambigua*: phytochemical and antimicrobial screening of the whole plant. *Ethnobotanical Leaflets*.

